# Comparison of avoidance assay techniques to determine the response to 1-octanol in *C. elegans*

**DOI:** 10.17912/micropub.biology.001177

**Published:** 2024-04-10

**Authors:** Olivia R. White, Bianca Graziano, Laura Bianchi

**Affiliations:** 1 Physiology and Biophysics, University of Miami Health System, Miami, Florida, United States

## Abstract

In *C. elegans* , avoidance behaviors are vital for the nematode's ability to respond to noxious environmental stimuli, including the odorant 1-octanol. To test avoidance to 1-octanol, researchers expose *C. elegans* to this odorant and determine the time taken to initiate backward locomotion. However, the 1-octanol avoidance assay is sensitive to sensory adaptation, where the avoidance response is reduced due to overexposure to the odorant. Here, we examined two methods to expose nematodes to 1-octanol, using an eyelash hair or a p10 pipette tip, to compare their susceptibility to cause sensory adaptation.

**Figure 1. The extent of avoidance of and adaptation to 1-octanol depends on the method of exposure to the odorant:**
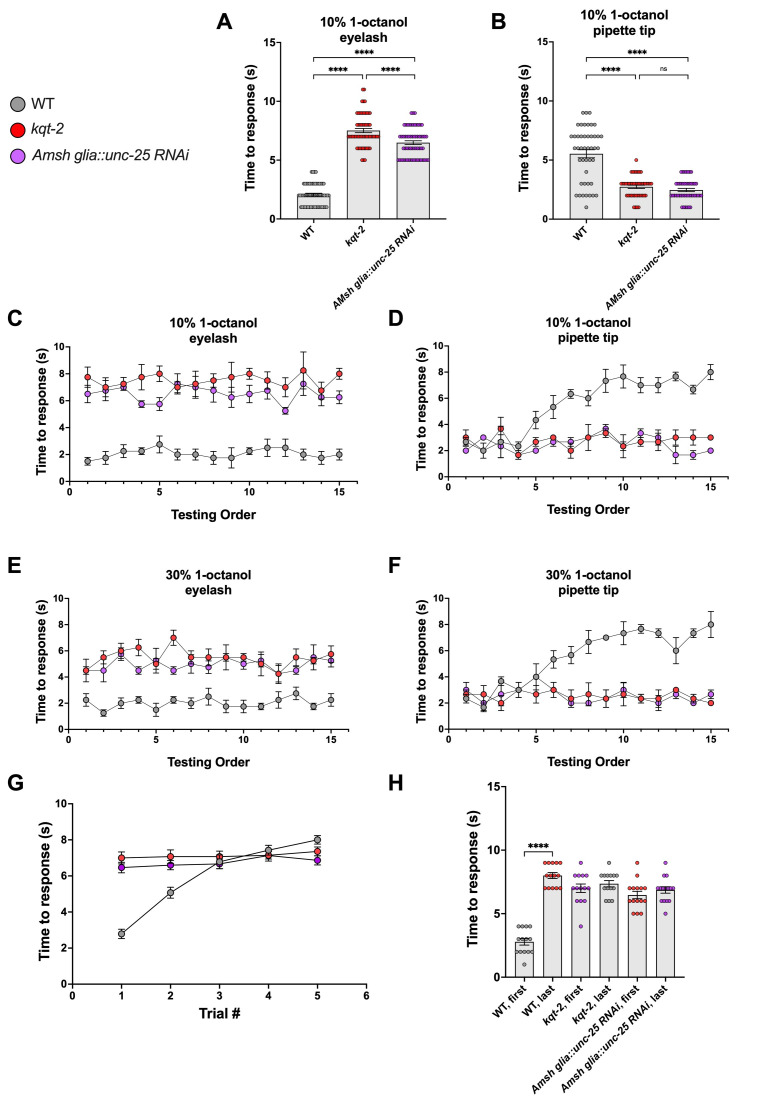
**(A)** Time to response to an eyelash hair dipped in 10% 1-octanol for wild type (WT), *kqt-2* knockout, and *unc-25* Amsh glia knock down. N = 60 each. **(B)** Same as in A but the nematodes were exposed to 10 μl of 10% 1-octanol contained in a p10 pipette tip. N = 45 each. **(C)** The time to response to 10% 1-octanol on an eyelash hair was plotted against the order in which each nematode was tested within the same plate for WT, *kqt-2* knockout, and *unc-25* Amsh glia knock down. Each point represents an average of 4 animals. **(D)** Same as in C, but the nematodes were exposed to 10 μl of 10% 1-octanol contained in a p10 pipette tip **.** Averages are from 4 animals each. **(E and F)** Same as in C and D, respectively, but using 30% 1-octanol. Each point represents the average of 4 animals. **(G)** Adaptation to repetitive exposures to 10% 1-octanol on an eyelash hair for WT, *kqt-2* knockout, and *unc-25* Amsh glia knock down. The time to response was determined following exposure of the same nematode to 1-octanol 5 consecutive times (# trials) with at least 30 seconds interval in between exposures. **(H)** The comparison of the time of response to 1-octanol in the first versus the fifth trials (data from G) are shown here for the same strains. N = 15, 14, and 15 for WT, *kqt-2* knockout, and *unc-25* Amsh glia knock down, respectively. Data are shown as individual data points and mean +/- SE in panels A, B, and H, and as mean +/- SE in panels C-G. Statistics were by ANOVA with Tukey correction, **** denotes p < 0.0001, ns stands for non-statistically significant.

## Description

In *C. elegans* , avoidance behaviors are vital for the nematode's ability to respond to noxious environmental stimuli. One of these behaviors is avoidance of the pungent odorant 1-octanol, which is mediated in large part by the ASH polymodal nociceptive neurons, a pair of sensory neurons located in the Amphid sensory organ in the nematode's head (Culotti and Russell, 1978;Bargmann et al., 1990;Kaplan and Horvitz, 1993;Troemel et al., 1995;Hart et al., 1999;Sambongi et al., 1999;Hilliard et al., 2002;Hilliard et al., 2004) . We and others have shown that the Amphid Sheath Glial cells (Amsh glia) are also needed for avoidance responses by releasing the neurotransmitter GABA. GABA, by interacting with GABA _A_ receptors LGC-38 in ASH neurons, dampens the excitability of these neurons thereby promoting avoidance behavior (Duan et al., 2020;Fernandez-Abascal et al., 2022;Wang et al., 2022;Graziano et al., 2024) . Thus, by measuring *C. elegans* avoidance responses to the repulsive odorant 1-octanol, we aim to decipher the principles of glia/neuron crosstalk.

To test 1-octanol avoidance, researchers use population or single worm assays (Troemel et al., 1995;Troemel et al., 1997) . In single worm assays, nematodes are exposed to the odorant by positioning, near the nose of a forward moving animal, either an eyelash hair or hair from a paint brush dipped in this odorant (“smell on a stick” assay) (Troemel et al., 1995;Chao et al., 2004) , a glass pipette dipped in the odorant (Williams et al., 2018) , or a p10 automatic pipette fitted with a tip containing 1-octanol (Duan et al., 2020) . The amount of 1-octanol used by the three methods is different, from nanoliters trapped on the hair in the “smell on a stick” assay to up to a few microliters in the method using the glass pipette or the p10 tip. Indeed, even simply dipping a glass capillary or a pipette tip in a solution causes the solution to enter these by capillarity. Different amounts of odorant are bound to have different effects on the air saturation with the odorant. Thus, given that normally 10 worms are placed on a plate for this assay, we postulated that there might be a difference in the avoidance and adaptation indexes depending on the method employed. More specifically, the time to response might be greater for the last worms tested in assays using the glass pipette or the p10 tip, versus the “smell on a stick” assay.

In these experiments, we compared the “smell on a stick” assay with the p10 tip method in which the tip of the pipette was filled with 10 μl of 1-octanol and tested three strains: WT, the knockout of the K ^+^ channel *kqt-2* , and the Amsh glial knockdown of the GABA synthetic enzyme *unc-25* (Duan et al., 2020;Fernandez-Abascal et al., 2022;Wang et al., 2022;Graziano et al., 2024) . Using the eyelash hair and 10% 1-octanol, we observed 1-octanol avoidance deficits in *kqt-2* knockout and *unc-25* Amsh glial knockdown nematodes. Indeed, while WT nematodes took ~2 s to respond to the odorant, *kqt-2* knockout and *unc-25* glial knockdown took ~6 s ( Fig. 1A ) (Wang et al., 2022;Graziano et al., 2024) . When we tested these strains using the p10 pipette method though, we found two major differences, 1) the time to avoidance of the WT nematodes was significantly higher and 2) the time to avoidance of *kqt-2* knockout and *unc-25* glial knockdown nematodes was lower as compared to the eyelash hair method ( Figure 1B ). Given that different amounts of odorant are used in these two assays and given that that 10 nematodes were present in the assay plate, we wondered whether differences in levels of air saturation with the odorant and the extent of adaptation to the odorant might, at least in part, explain the discrepancy in these results. To test this idea, we monitored the order in which the nematodes were tested and plotted their avoidance response times individually. By using the eyelash hair to expose the nematodes to 1-octanol, we found that all three nematode strains had relatively consistent avoidance response times throughout the duration of the assay ( Figure 1C ). On the contrary, when using the p10 pipette tip method, WT worms became less sensitive to the odorant the later they were tested in the assay, suggesting that they experienced adaptation to the odorant as they waited to be tested. Strikingly though, *kqt-2* knockout and *unc-25* glial knockdown nematodes retained the same sensitivity to the odorant throughout the assay using the pipette without signs of adaptation ( Fig. 1D ).

To establish whether the 1-octanol dilution would influence the avoidance response, we repeated the experiments using 30% 1-octanol. Overall, we obtained similar results, though with the eyelash method *kqt-2* knockout and *unc-25* glial knockdown nematodes had shorter times to response compared to 10% 1-octanol ( Fig. 1E and F ). Indeed, their times to response were more like the ones obtained using the pipette tip method, underscoring that the pipette tip method exposes animals to higher amount of odorant. Taken together, these results show that the p10 pipette tip method is more prone to cause adaptation to the odorant regardless of the dilution. These results also show that adaptation to the odorant may in part explain the difference in the results obtained using the eyelash hair versus the p10 pipette tip method ( Fig. 1 A and B ).

To directly establish whether sensory adaptation to 1-octanol could mimic data obtained using the p10 pipette tip method, we exposed nematodes on individual plates to the odorant using the eyelash hair 5 consecutive times and recorded the avoidance time in each trial. We found that WT nematodes displayed significant adaption to 1-octanol as compared to the *kqt-2* knockout and *unc-25* glial knockdown nematodes, with an average time to response like the one seen with the p10 pipette tip method ( Figure 1G and H, 6.01 +/- 2.11 s versus Figure B, 5.53 +/- 2.30 s). Interestingly though, when using the eyelash hair, *kqt-2* knockout and *unc-25* glial knockdown nematodes still took ~6 s to respond. This is in contrast with the p10 pipette tip method in which these two strains respond in ~ 2 s. Together, these data support the idea that with the p10 pipette tip method the nematodes are exposed to an overall higher concentration of the odorant and for a longer period of time.

We conclude that both these methods were useful in revealing sensory deficits in *kqt-2* knockout and *unc-25* glial knockdown nematodes, by highlighting the reduced sensitivity to the odorant (eyelash hair method) and the loss of adaptation (p10 pipette tip method). However, our analysis emphasizes the need to control environmental exposure to chemosensory stimuli in avoidance assays. For example, if the p10 pipette tip method is used, nematodes should be placed in individual plates and kept away from the assay area to prevent adaptation. Furthermore, a consistent amount of odorant in the tip should be used to allow comparisons of data across experiments. A combination of these two methods might be a useful tool to determine whether mutants have altered sensitivity or adaptation to the odorant, or both.

## Methods

*Strains and strain maintenance:*

The nematodes were kept at 20°C on nematode growth medium (NGM) agar plates seeded with the OP50 *Escherichia coli* strain (Brenner, 1974) . All the experiments were conducted on one day old adult nematodes. Age-synchronized populations were obtained by picking L4 larvae and isolating them on fresh NGM agar plates for about 18 hours.

*Behavioral Assays:*

The 1-octanol avoidance assays were performed as previously published with some modifications (Troemel et al., 1995;Chao et al., 2004) . The morning before conducting the assay, 40 μl of OP50 were seeded on 60 mm NGM plates and allow to dry for at least 30 minutes at room temperature. Fifteen to 20 one-day-old nematodes were transferred onto the seeded NGM assay plates and the researcher conducting the assay was blinded. The nematodes were left to acclimate to the new plate conditions for approximately 30 to 45 minutes. In the meantime, 1-octanol was diluted to 10% or 30% in 100% ethanol. 40 μl of diluted 1-octanol was aliquoted into separate PCR tubes for the experiments. The 1-octanol aliquots were kept sealed and a fair distance away from the nematodes when assessing 1-octanol avoidance. For the experiments using an eyelash, an eyelash was glued onto the tip of a toothpick. The eyelash was saturated in the diluted 1-octanol, avoiding the tip of the toothpick. Then, the eyelash was placed in front of a forward-moving nematode. The 1-octanol avoidance response time was recorded upon initiation of backward locomotion. For subsequent trials, the eyelash was re-saturated in diluted 1-octanol following the recording of avoidance time to ensure the presence of the odorant throughout the assay. For the experiments using a 10 μl pipette tip, the pipette tip was filled with 10 μl of diluted 1-octanol and placed in front of a forward-moving nematode. The pipette tip was temporarily removed from the vicinity of the NGM plate to record the avoidance times before it was reintroduced to an untested nematode. When assessing 1-octanol adaptation, the “smell on a stick assay” was used and each nematode was tested a total of 5 times with at least 30 seconds interval between trails. For these assays, we picked one worm per plate and used 35 mm instead of 60 mm plates. Thirty five mm plates were seeded with 20 μl of OP50 .

## Reagents

**Table:** 

**STRAIN**	**GENOTYPE**	**AVAILABLE FROM**
Bacterial and virus strains		
*OP50*	*Escherichia coli*	CGC
Strains		
*N* 2	*Caenorhabditis elegans*	CGC
RB883	*kqt-2 ( ok732 ) *	CGC
BLC500	*Ex[PT02B11* . *3* :: *unc-25 RNAi + unc-122p* :: *GFP]*	(Wang et al., 2022) (Fernandez-Abascal et al., 2022;Graziano et al., 2024)

## References

[R1] Bargmann CI, Thomas JH, Horvitz HR (1990). Chemosensory cell function in the behavior and development of Caenorhabditis elegans.. Cold Spring Harb Symp Quant Biol.

[R2] Brenner S (1974). The genetics of Caenorhabditis elegans.. Genetics.

[R3] Chao MY, Komatsu H, Fukuto HS, Dionne HM, Hart AC (2004). Feeding status and serotonin rapidly and reversibly modulate a Caenorhabditis elegans chemosensory circuit.. Proc Natl Acad Sci U S A.

[R4] Culotti JG, Russell RL (1978). Osmotic avoidance defective mutants of the nematode Caenorhabditis elegans.. Genetics.

[R5] Duan D, Zhang H, Yue X, Fan Y, Xue Y, Shao J, Ding G, Chen D, Li S, Cheng H, Zhang X, Zou W, Liu J, Zhao J, Wang L, Zhao B, Wang Z, Xu S, Wen Q, Liu J, Duan S, Kang L (2020). Sensory Glia Detect Repulsive Odorants and Drive Olfactory Adaptation.. Neuron.

[R6] Fernandez-Abascal J, Johnson CK, Graziano B, Wang L, Encalada N, Bianchi L (2021). A glial ClC Cl
^-^
channel mediates nose touch responses in C.&nbsp;elegans.. Neuron.

[R7] Graziano B, Wang L, White OR, Kaplan DH, Fernandez-Abascal J, Bianchi L (2024). Glial KCNQ K(+) channels control neuronal output by regulating GABA release from glia in C.&nbsp;elegans.. Neuron.

[R8] Hart AC, Kass J, Shapiro JE, Kaplan JM (1999). Distinct signaling pathways mediate touch and osmosensory responses in a polymodal sensory neuron.. J Neurosci.

[R9] Hilliard MA, Bargmann CI, Bazzicalupo P (2002). C. elegans responds to chemical repellents by integrating sensory inputs from the head and the tail.. Curr Biol.

[R10] Hilliard MA, Bergamasco C, Arbucci S, Plasterk RH, Bazzicalupo P (2004). Worms taste bitter: ASH neurons, QUI-1, GPA-3 and ODR-3 mediate quinine avoidance in Caenorhabditis elegans.. EMBO J.

[R11] Kaplan JM, Horvitz HR (1993). A dual mechanosensory and chemosensory neuron in Caenorhabditis elegans.. Proc Natl Acad Sci U S A.

[R12] Sambongi Y, Nagae T, Liu Y, Yoshimizu T, Takeda K, Wada Y, Futai M (1999). Sensing of cadmium and copper ions by externally exposed ADL, ASE, and ASH neurons elicits avoidance response in Caenorhabditis elegans.. Neuroreport.

[R13] Troemel ER, Chou JH, Dwyer ND, Colbert HA, Bargmann CI (1995). Divergent seven transmembrane receptors are candidate chemosensory receptors in C. elegans.. Cell.

[R14] Troemel ER, Kimmel BE, Bargmann CI (1997). Reprogramming chemotaxis responses: sensory neurons define olfactory preferences in C. elegans.. Cell.

[R15] Wang L, Graziano B, Encalada N, Fernandez-Abascal J, Kaplan DH, Bianchi L (2022). Glial regulators of ions and solutes required for specific chemosensory functions in Caenorhabditis elegans.. iScience.

[R16] Williams PDE, Zahratka JA, Rodenbeck M, Wanamaker J, Linzie H, Bamber BA (2018). Serotonin Disinhibits a Caenorhabditis elegans Sensory Neuron by Suppressing Ca(2+)-Dependent Negative Feedback.. J Neurosci.

